# RBS Depth Profiling Analysis of (Ti, Al)N/MoN and CrN/MoN Multilayers

**DOI:** 10.1186/s11671-017-1921-3

**Published:** 2017-03-01

**Authors:** Bin Han, Zesong Wang, Neena Devi, K. K. Kondamareddy, Zhenguo Wang, Na Li, Wenbin Zuo, Dejun Fu, Chuansheng Liu

**Affiliations:** 1 Key Laboratory of Artificial Micro- and Nano-Materials of Ministry of Education and School of Physics and Technology, Wuhan University, Wuhan, 430072 China; 2 Hubei Nuclear Solid Physics Key Laboratory at School of Physics and Technology, Wuhan University, Wuhan, 430072 China

**Keywords:** RBS, MoN, Multilayer, Microstructure, Depth profiling

## Abstract

(Ti, Al)N/MoN and CrN/MoN multilayered films were synthesized on Si (100) surface by multi-cathodic arc ion plating system with various bilayer periods. The elemental composition and depth profiling of the films were investigated by Rutherford backscattering spectroscopy (RBS) using 2.42 and 1.52 MeV Li^2+^ ion beams and different incident angles (0°, 15°, 37°, and 53°). The microstructures of (Ti, Al)N/MoN multilayered films were evaluated by X-ray diffraction. The multilayer periods and thickness of the multilayered films were characterized by scanning electron microscopy (SEM) and high-resolution transmission electron microscopy (HR-TEM) and then compared with RBS results.

## Background

Multilayered, multicomponent, and nanostructured films are widely used in modern material engineering for their great contributions to improving protective properties of versatile industrial products involving hardness, wear, and corrosion resistance, oxidation resistance at high temperature [1–5]. Generally, a multilayered film containing two alternating sublayers has a significant parameter of modulation period defined as the thickness of a bilayer at nanoscale. Among the multilayer-film family, hard nitride-based coatings as one of the most prospective functional materials are so attractive that have been exploring by means of the optimized preparation processes and novel analytical techniques [6–9]. It has already been proved that the multilayered coatings have better properties compared with the monolayers [10–12] because the combination of two kinds of coatings can provide superior performance for the cutting tools and the thickness of the sublayer inlayed in the multilayered structure plays a significant role for vigorous properties of nano-composite coatings [13, 14]. It is necessary to adopt appropriate methods to fabricate and probe new multilayered structure films for further industrial application.

MoN films are remarkable for the self-lubrication over a wide temperature range, which leads to a low-coefficient friction and low wear rate [15–17]. The excellent tribological signatures are introduced by the formation of lubricious oxides, such as MoO_3_ demonstrated by Koshy [18]. CrN coatings can exhibit the extraordinary oxidation, wear, and corrosion resistance [19, 20] while has rather high-friction coefficient (0.4–0.8 in air) [21–23]. Fabrication of CrN/Mo_2_N multilayered structure is an effective route to decrease the friction coefficient of bearing coatings like CrN from 0.6–0.8 to 0.3–0.4 at room temperature. TiAlN coatings are usually used as the cutting tools, wear protections, and contact materials in the microelectronics due to its high hardness, chemical inertness, and thermodynamic stability [24–28]. Addition of Mo to TiAlN forming TiAlN/MoN multilayers or nanocomposites can diminish the friction coefficient ranging from 0.8–0.9 to 0.3–0.4 at higher temperatures [29, 30]. The super hardness in nano-composite thin films is obtained when the small crystallites are detached by a discrepant boundary with the high cohesive strength (Patscheider et al.) [31]. Briefly, it is extremely important and useful to analyze the multilayered structure consisting of MoN, CrN as well as other functional interlayers in ion plating applications.

Rutherford backscattering spectrometry (RBS) based on elastic collision has been utilized as a conventional tool for analysis of the thin film or the solid compound comprised intrinsic elements with enormous mass difference [32–34]. It is determinate to act as one of the most efficient non-destructive techniques for the depth profiling among nanoscale characterizations for the thin film, which can evaluate the relative atomic concentration as a function of depth at a unit of the areal density. It can also give the evidence on the atomic diffusion located at the interface of two individual layers as depth profiling in the film resulted from annealing. Usually, cross-section scanning electron microscopy (SEM) and transmission electron microscopy (TEM) are employed to probe the microstructure feature of the multilayered films. However, the destructiveness and complication of accurate sampling detected by these methods have to take into consideration. By contrast, RBS is certainly a better alternative for the composition and depth profiling due to its significant advantage and quantification for both the thin films and bulk materials [35, 36].

To some extent, the systemic resolution of RBS measurement depends on the incident ion species, initial energy, and energy resolution of detector besides vacuum degree for beam transportation. Frequently, selecting proton as incident ion has better sensitivity for light element detection than ^4^He^2+^ or other heavy ions ascribed to its great penetrability and smaller straggling, but it is not sensitive for ultrathin film as the multilayered films consisting of number of sublayers at a thickness of several nanometers. In order to obtain more reasonable detection results, a heavier incoming MeV ion is probable to contribute much better mass resolution and depth resolution instead of energy resolution.

In this paper, we have used 2.42 and 1.52 MeV ^7^Li^2+^ ion beams as projectile and different incident angles (0°, 15°, 37°, and 53°) to study the structure of (Ti, Al)N/MoN and CrN/MoN multilayered films. X-ray diffraction (XRD) was employed to probe the microstructure of (Ti, Al)N/MoN. The element composition of CrN/MoN multilayered films was measured by Sirion FEG SEM with EDAX genesis 7000 EDS. X-ray photoelectron spectra (XPS, XSAM800 KRATOS) collected by Thermo Scientific Escalab 250 Xi spectrometer were used to confirm the elemental composition of (Ti, Al)N/MoN. At the same time, SEM and cross-sectional high resolution TEM (HRTEM) were also used to measure their modulation periods comparing with the results of RBS.

## Methods

The (Ti, Al)N/MoN multilayered films were deposited on the polished Si(100) with Ti_0.7_Al_0.3_ and Mo targets by the cathodic arc plasma deposition system whose configuration was detailed in our previous work [37]. During the deposition process, the nitrogen gas was fed into the chamber and the deposition pressure kept at 2.5 Pa while bias voltage of the substrate was −300 V. The (Ti, Al)N/MoN films with different modulation periods were fabricated by varying the substrate rotation speed (SRS) from 1 to 3 rpm. Similarly, the CrN/MoN multilayered films were deposited on the Si (100) substrates using Cr and Mo metal targets with a purity of more than 99.95%. Prior to deposition, the substrates were cleaned by a standard technique using ultrasonic degreasing and exposed to bombardment of Cr^+^ ions at −800 V for 10 min so as to remove the surface contaminants and reduce the roughness. After feeding reactive nitrogen gas to deposition process continuously, the vacuum and negative bias were about 2.0 Pa and 200 V, respectively. To achieve the multilayered films with various modulation periods, SRS was also varied from 2 to 6 rpm.

**Fig. 1:**
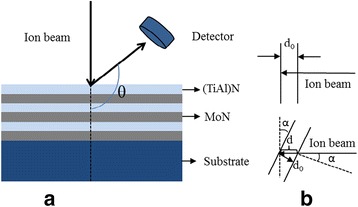
Schematic diagram of **a** (Ti, Al)N/MoN multilayered films deposited on Si substrate and **b** the relation between incident angle and tilt angle of thin film

RBS was introduced using 2.42 and 1.52 MeV Li^2+^ ion beams as projectiles to study the structures of two kinds of multilayered films. Figure 1a gives schematic diagram of (Ti, Al)N/MoN multilayered films deposited on Si substrate. The measurements were carried out by means of the double 1.7 MV Tandetron accelerator (Ionx, GIC 4117), where the ion current was about 5 nA impinging on the target with a flux of 5 μC monitored with a beam integrator and the diameter of a circular beam spot was 1.5 mm. The scattered ions were acquired by a silicon surface barrier detector at a resolution of 15 keV for ^4^He^2+^ ions mounted at 170° of backscattering angle under 5 × 10^−4^ Pa. RBS spectra were fitted by using the SIMNRA code [38]. Furthermore, the angle of incidence of ion beam α which can be expressed as an angle between the incident ion beams extended line and the normal of top surface of the sample is equal to the tilt angle of the sample when the direction of incident ion beam is fixed, as shown in Fig. 1b. By tilting the sample, i.e., changing the incident angle α can enhance the detecting depth of the sample. Simply, the detected depth *d* = *d*_0_/cos*α*, where, *d*_0_ is a certain depth in the sample when *α* = 0°. The structure, surface topography, and element composition of the films were analyzed by using XRD (D_8_ advanced) with a Cu Kα radiation and Sirion FEG SEM with EDAX genesis 7000 EDS. X-ray photoelectron spectra (XPS, XSAM800 KRATOS) collected by Thermo Scientific Escalab 250 Xi spectrometer were used to confirm the elemental composition of (Ti, Al)N/MoN. The cross-sectional HR-TEM images were also displayed to illustrate the microstructure of the samples at JEM 2010 FEF (UHR) operated at 200 kV.

## Results and Discussion

**Fig. 2:**
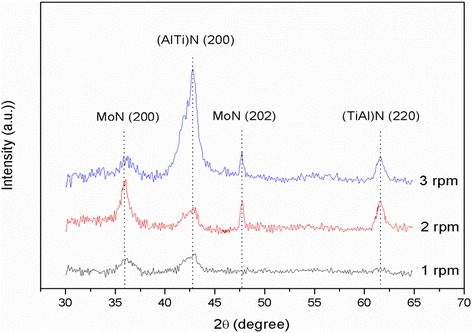
X-ray diffraction patterns of (Ti, Al)N/MoN multilayered films at a SRS of 1, 2, and 3 rpm

The microstructures of (Ti, Al)N/MoN multilayered films at a SRS of 1, 2, and 3 rpm were measured in Fig. 2. There are crystallographic orientations (200) and (202) in MoN sublayers which are very likely to imply the growth of individual film along these preferred orientations during physical vapor deposition. The diffraction peaks (200) at 42.9° and (220) at 62° are assigned to face-centered cubic AlN and TiN, respectively, and these peaks shifted to higher angles in comparison with corresponding standard indicator on TiN (Fm-3 m space group, PDF-65-0970).

This subtle phase angle shift may be ascribed to the reduction of lattice parameter which is probable to be explained by smaller interstitial Al^3+^ ions replacing Ti^3+^ ions. It is concluded that the bilayer in films has no influence on the phase formation at the different SRSs but can interact on grain sizes of crystals in the sublayers.

**Fig. 3:**
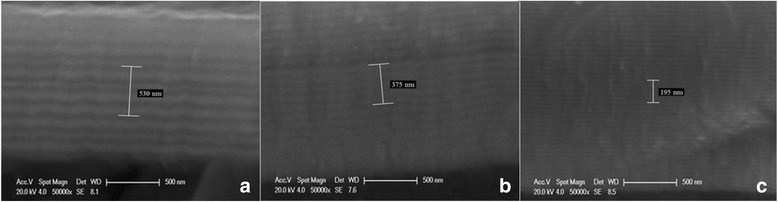
SEM images of (Ti, Al)N/MoN multilayered films at a SRS of 1, 2, and 3 rpm

SEM images of multilayered films at 1, 2, and 3 rpm are displayed in Fig. 3. It can be estimated that the darker layer is assigned to MoN and another is (Ti, Al)N. The mean thickness of bilayers can be determined at approximately 106, 75, and 39 nm, also indicating their differential modulation periods. It is noted that a mass of periodically alternative striations deposited on the surface of the matrix not only can validate the crystallized MoN and (Ti, Al)N as compared to XRD results but also can give a tendency that a thinner and more uniform multilayered film was fabricated at a higher revolutions per minute by expanding contrast between the multilayered film and the matrix.

**Fig. 4:**
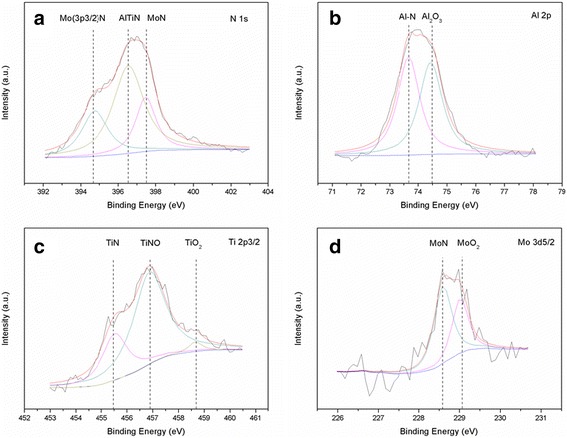
XPS spectra of the (Ti, Al)N/MoN multilayer deposited at 2 rpm. (**a**) N1s, (**b**) Al 2p, (**c**) Ti 2p3/2, (**d**) Mo 3d5/2

Figure 4 presents XPS core level spectra of the sample on Si at 2 rpm, which shows the composition of the (Ti, Al)N/MoN multilayer. The binding energy values are corrected relatively to the C1s level at 284.6 eV. Gaussian-Lorentzian functions were used for fitting the spectra. Figure 4a shows that N 1s peak can be deconvolved into three peaks at 394.7, 396.7, and 397.6 eV. One of N 1s peak is observed near Mo 3p3/2 peak and overlaps it partially at 394.7 eV that match with the literature data for MoN [39]. Figure 4b shows that Al 2p3/2 peak is positioned at 74.45 eV and corresponds to Al_2_O_3_ [40]. Figure 4c shows that Ti 2p3/2 spectra were deconvolved into three peaks at 455.4, 457.0, and 458.9 eV, which are usually associated with the presence of TiN, Ti_2_O_3_ or TiNO, and TiO_2_, respectively, [41, 42] suggesting moderate surface oxidation. Figure 4d shows that Mo 3d5/2 peak at 228.6 eV corresponds to Mo–N bonding [43, 44]. Asymmetry of the Mo3d doublet assumes the presence of a peak at 229.1 eV for 3d5/2 peak and corresponds to surface MoO_2_ [45, 46]. In conclusion, XPS measurement shows the presence of Al–N, Ti–N, and Mo–N bonding corresponding to AlTiN and MoN.

**Fig. 5:**
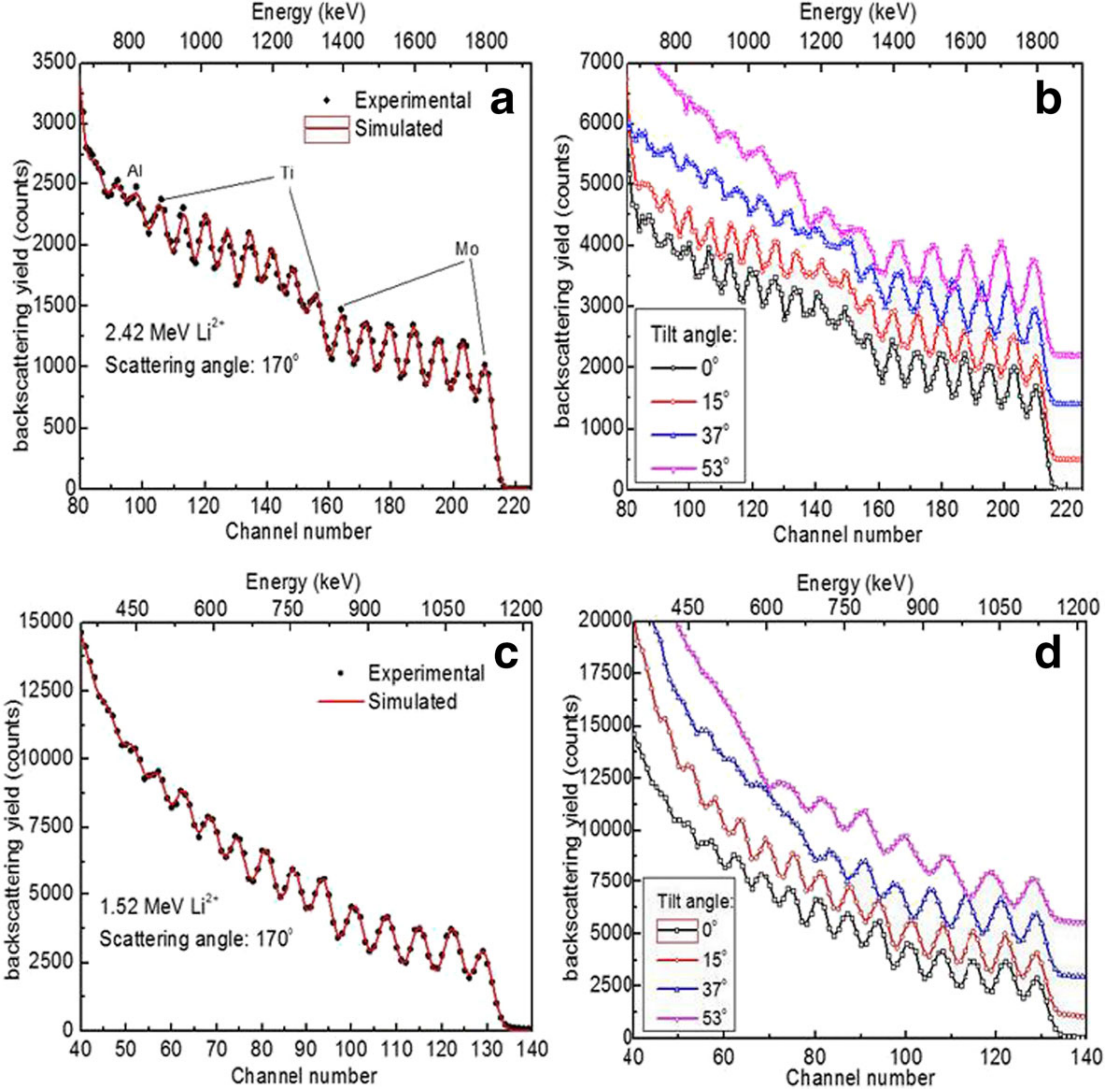
RBS spectra of (Ti, Al)N/MoN multilayer at 2 rpm through incident 2.42 and 1.52 MeV Li^2+^ ions at 170°. **a** Initial energy *E*_0_ = 2.42 MeV, the angle of incidence *α* = 0°. **b** *E*_0_ = 2.42 MeV, *α* = 0°, 15°, 37°, and 53. **c** *E*_0_ = 1.52 MeV, *α* = 0°, and **d** *E*_0_ = 1.52 MeV, *α* = 0°, 15°, 37°, and 53°

The quantitative measurements of Mo, Ti, and Al are implemented by RBS with 2.42 MeV Li^2+^ ion beams impinging vertically on the (Ti, Al)N/MoN multilayer at 2 rpm, as shown in Fig. 5a, where discrete solid dots represent experimental data and the red curve is simulated results from SIMNRA, and the stopping power data by Ziegler and Biersack have been applied in simulation. Mo in MoN and Ti, Al in (Ti, Al)N can be addressed obviously while the position of N is not depicted due to its smaller scattering cross section at low-energy regions. Fortunately, almost perfect fitting data can be given in SIMNRA code when simulated results agree well with the experimental ones. The thickness is calculated from the following formula:

$$ d=\frac{ A M}{\rho {N}_A} $$

where, *d* is the thickness, *A* is the area density, *M* is the molecular mass, *ρ* is bulk density, and *N*_*A*_ is Avogadro constant. The results indicate that the area density of a single MoN layer ranges from 1.7 × 10^17^ atoms/cm^2^ to 2.7 × 10^17^ atoms/cm^2^ while the area density of (Ti, Al)N is 1.5 × 10^17^ atoms/cm^2^ ~ 2.0 × 10^17^ atoms/cm^2^. It is assumed that the bulk density of MoN is 9.05 g/cm^3^ and Ti_0.7_Al_0.3_N is 5.0 g/cm^3^. The average thicknesses of two sublayers were approximately 44 and 32 nm, summed 76 nm of the modulation period at 2 rpm that can match very well with SEM result. It is confirmed when *α* increases to 15°, 37°, and 53° (Fig. 5b), the scattering cross-section peaks of Mo, Ti, and Al can be broadened gradually that implies the detected resolution at a certain depth is improved. Whereas, a greater detected depth resulting in a severer energy straggling can weaken the systemic energy resolution, which can be interpreted that the signal peaks of Ti and Al at low-energy region are broadened more seriously.

Substantially, the classic Rutherford backscattering cross section *σ*_*R*_ can be expressed as:

$$ {\sigma}_R={\left(\frac{Z_1{Z}_2{\mathrm{e}}^2}{2 E}\right)}^2\cdot \frac{{\left[{\left({M_2}^2-{M_1}^2{ \sin}^2\theta \right)}^{1/2}+{M}_2 \cos \theta \right]}^2}{M_2{ \sin}^4\theta {\left({M_2}^2-{M_1}^2{ \sin}^2\theta \right)}^{1/2}} $$

where, *Z1*, *Z2* is atomic number of the incident ion and the target atom, *M1*, *M2* is their relative atomic mass, and *E* and *θ* are corresponding to incident ion energy and backscattering angle, respectively [47]. Decreasing the initial energy *E*_0_, the backscattering cross section is increased that can dedicate better mass differences to target atoms in the sample. In Fig. 5c, when *E*_0_ is reduced from 2.42 to 1.52 MeV, the backscattering yields of all the elements have increased to five times higher than that of 2.42 MeV. A straightforward variety is that visible signal peaks from neighbor-surface sublayers reduce, such as from 7 to 5 peaks for Mo, revealing a shallower detected depth in the same condition. Comparatively, the fitting data give a thickness of 1.9 × 10^17^ ~ 2.8 × 10^17^ atoms/cm^2^ for monolayer MoN and 1.15 × 10^17^ ~ 1.8 × 10^17^ atoms/cm^2^ for monolayer (Ti, Al)N, which is corresponding to an average thickness of 47.5 and 34.5 nm, respectively. When the angle of incidence changes from 0° to 53° (Fig. 5d), the effective thickness of the outmost monolayer film is increased intensely while the sublayers located in deeper positions have worse energy resolution, especially for larger tilt angle 37° and 53°. It is concluded that incident ion beam with low energy can lead to a relative shallower detected depth but can be beneficial to detect ultrathin film beneath 10 nm.

**Fig. 6:**
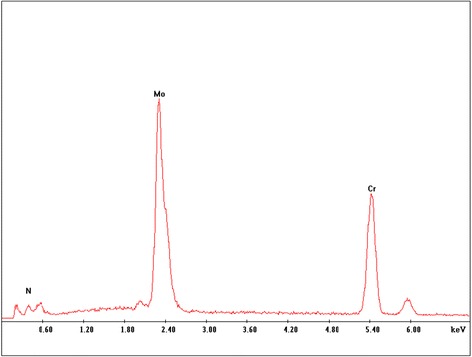
The EDS spectra of CrN/MoN multilayered film deposited at 2 rpm

Another MoN-based multilayered film CrN/MoN was characterized by RBS via 1.52 MeV Li^2+^ ions impinging on the surface perpendicularly. Figure 6 shows the EDS spectra of CrN/MoN at SRS of 2 rpm. The atomic concentrations of Mo, Cr, and N are 26.7, 34, and 39.3 at .%, respectively. All multilayered films have similar elemental composition.

**Fig. 7:**
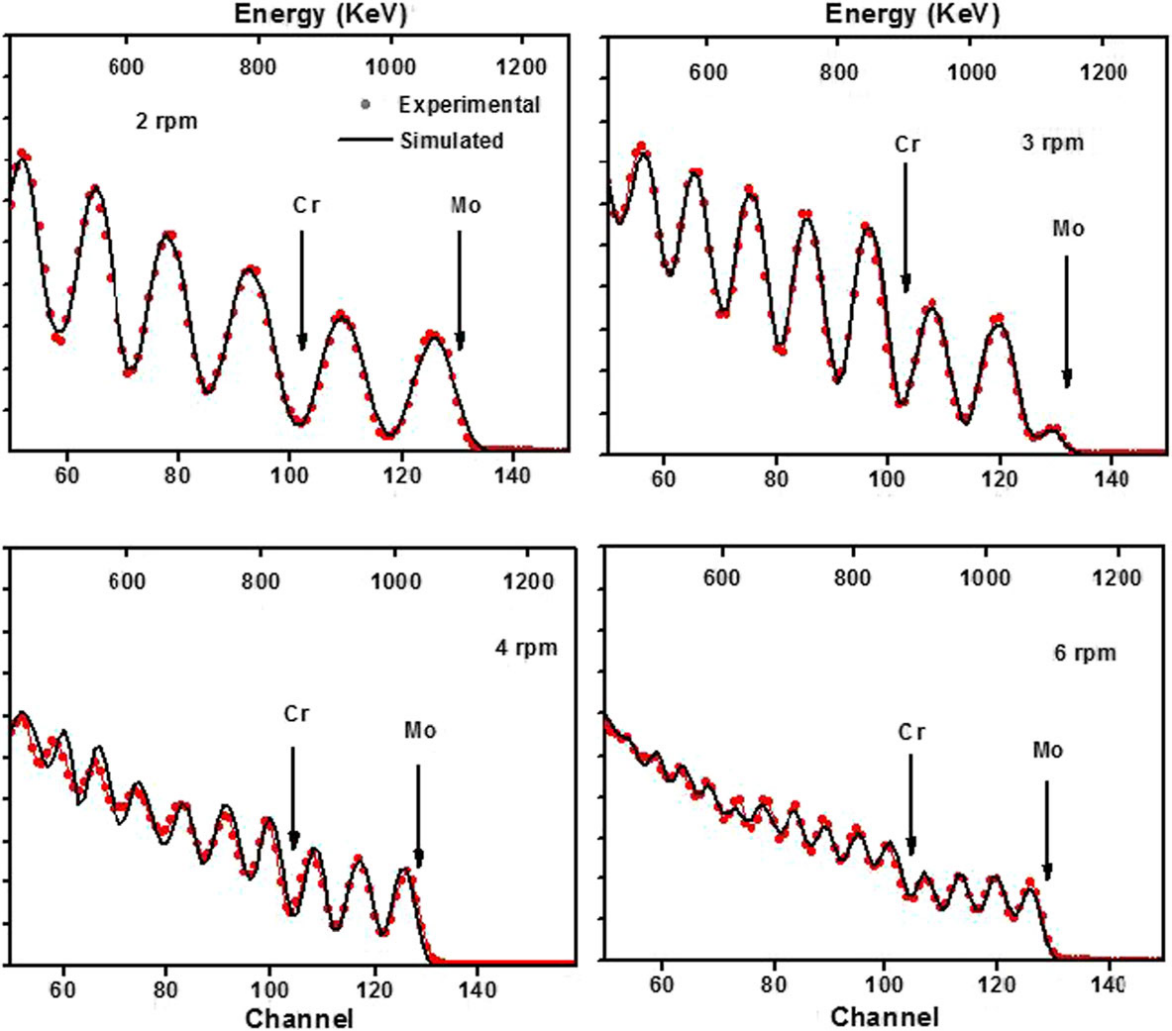
The experimental and simulated RBS spectra of CrN/MoN multilayered films at SRS of 2, 3, 4, and 6 rpm

Figure 7 illustrates RBS spectra of CrN/MoN at 2, 3, 4, and 6 rpm. The thickness of individual sublayer strongly depends on SRS. Low-speed deposition can get much thicker film. Therefore, visible signal peak numbers of Mo and Cr increase as the increasement of SRS when energy losses are refrained from the same interval, such as from 1100 to 600 keV. The statistical backscattering yields diminish which can be attributed to lower collision probabilities of few target atoms in films deposited at a larger speed. The surface peak intensity of Mo in the outmost MoN at 3 rpm is rather lower ascribed to the deposition process that was paused during the MoN sublayer growth at a certain time. With respect to quantification on the thickness of individual sublayer, a bulk density of 9.05 g/cm^3^ for MoN and 5.9 g/cm^3^ for CrN were introduced to figure out it at nanometer combined with fitting data from SIMNRA code. The modulation periods of CrN/MoN multilayered films were evaluated at 69.9, 49.6, 39.8, and 18.5 nm at 2, 3, 4 and 6 rpm, respectively. At the same time, the minimum monolayer of CrN is 8.5 nm and MoN is 10 nm which indicated our RBS characterization has a relative good depth profiling [48, 49].

**Fig. 8:**
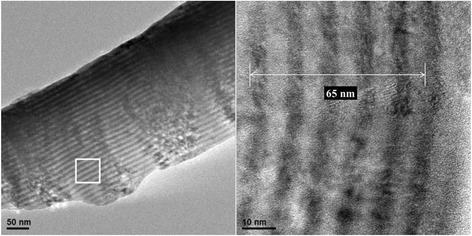
Cross-sectional HRTEM micrograph of CrN/MoN multilayered film at 6 rpm

The cross-sectional HR-TEM micrograph of CrN/MoN multilayered film at 6 rpm is shown in Fig. 8. The average modulation period at 6 rpm is estimated at 13 nm which is less than 18.5 nm of RBS result. A possible explanation is that there are few microcrystalline layers embedded in massive amorphous layers for the multilayered film at a higher rpm, HR-TEM has a better depth-profiling resolution at a restricted region as compared to RBS characterization. It is mandatory for SIMNRA simulation program to count a given element content in thin film as the following formula:

$$ Y={\sigma}_R\cdot \varOmega \cdot Q\cdot \rho \cdot {d}_0/ \cos \alpha $$

where, *Y* is yield of backscattered ions, *σ*_*R*_ is Rutherford backscattering cross section, *Q* is total number of incident ion charges, *ρ* is bulk density of target sample, and *d*_0_/cos *α* is the detected depth when the incident angel is *α*. The fitting data from SIMNRA give the areal density *ρ* ⋅ *d*_0_/cos *α* at atoms/cm^2^ (unit) [47]. The thickness (nm) is proportional to the areal density and molecular mass of compound. However, during quantitative RBS measurements via fitting data of CrN/MoN, the areal density and molecular mass of homogenous compound instead of hybridized structures consisting of single phase compound and amorphous phase mixtures were taken into inconsideration that can lead to calculated value is larger than the actual value.

## Conclusions

We have analyzed (Ti, Al)N/MoN and CrN/MoN multilayered films on Si substrate by using 1.52 and 2.42 MeV Li^2+^ ion beams of RBS. The results demonstrated that the 1.52 MeV ion beam is superior in depth resolution, whereas the 2.42 MeV ion beam is advantageous for deeper path detection. It is seen that SIMNRA simulation data agree well with the SEM results of (Ti, Al)N/MoN films at 2 rpm. The scattering cross-section peaks were broadened gradually with increases in the angle of incidence (*α*) of ion beam which implies the improvement in the detected resolution at a certain depth. The monolayers of CrN and MoN are 8.5 and 10 nm, respectively, when the film has smallest bilayer period 18.5 nm which provided that a relative good depth resolution about 10 nm. Finally, RBS depth profiling proved to be a useful structural tool to evaluate the multilayer structure and chemical composition.
